# A Physiology-Based Seizure Detection System for Multichannel EEG

**DOI:** 10.1371/journal.pone.0065862

**Published:** 2013-06-14

**Authors:** Chia-Ping Shen, Shih-Ting Liu, Wei-Zhi Zhou, Feng-Seng Lin, Andy Yan-Yu Lam, Hsiao-Ya Sung, Wei Chen, Jeng-Wei Lin, Ming-Jang Chiu, Ming-Kai Pan, Jui-Hung Kao, Jin-Ming Wu, Feipei Lai

**Affiliations:** 1 Graduate Institute of Biomedical Electronics and Bioinformatics, National Taiwan University, Taipei, Taiwan; 2 Department of Computer Science and Information Engineering, National Taiwan University, Taipei, Taiwan; 3 Department of Information Management, Tunghai University, Tai-Chung, Taiwan; 4 Department of Neurology, National Taiwan University Hospital, College of Medicine, National Taiwan University, Taipei, Taiwan; 5 Graduate Institute of Brain and Mind Sciences, National Taiwan University, Taipei, Taiwan; 6 Department of Psychology, College of Science, National Taiwan University, Taipei, Taiwan; University of Michigan, United States of America

## Abstract

**Background:**

Epilepsy is a common chronic neurological disorder characterized by recurrent unprovoked seizures. Electroencephalogram (EEG) signals play a critical role in the diagnosis of epilepsy. Multichannel EEGs contain more information than do single-channel EEGs. Automatic detection algorithms for spikes or seizures have traditionally been implemented on single-channel EEG, and algorithms for multichannel EEG are unavailable.

**Methodology:**

This study proposes a physiology-based detection system for epileptic seizures that uses multichannel EEG signals. The proposed technique was tested on two EEG data sets acquired from 18 patients. Both unipolar and bipolar EEG signals were analyzed. We employed sample entropy (*SampEn*), statistical values, and concepts used in clinical neurophysiology (e.g., phase reversals and potential fields of a bipolar EEG) to extract the features. We further tested the performance of a genetic algorithm cascaded with a support vector machine and post-classification spike matching.

**Principal Findings:**

We obtained 86.69% spike detection and 99.77% seizure detection for Data Set I. The detection system was further validated using the model trained by Data Set I on Data Set II. The system again showed high performance, with 91.18% detection of spikes and 99.22% seizure detection.

**Conclusion:**

We report a de novo EEG classification system for seizure and spike detection on multichannel EEG that includes physiology-based knowledge to enhance the performance of this type of system.

## Introduction

Electroencephalography (EEG) detects the most critical physiological signal in neurological practice. EEGs are obtained by placing electrodes on various positions of the scalp. EEG measures cerebral electrical activity and can detect epileptic seizures in patients with epilepsy, which afflicts approximately 1% of the population [1]. Patients with epilepsy frequently present features in their EEG electrical potentials that are of significant diagnostic importance, such as spikes or sharp waves. Routine EEG, which shows temporal and spatial information regarding the electrical activity of the brain, is frequently used to diagnose, monitor, and localize epileptogenic foci [2]. EEG is the gold standard for the classification of seizure types and the diagnosis of epileptic disorders.

EEG signals are frequently displayed in one of four manners: unipolar, bipolar, Laplacian, and average reference montage. We used both unipolar and bipolar montage to display epileptiform discharges in different locations. In a unipolar montage, both common references and ground references are used, and a single channel represents the electrical activity of a brain in a particular recording site. A bipolar EEG montage measures the potential difference between pairs of electrodes and is calculated by subtracting one unipolar measurement from another unipolar measurement, typically neighboring ones. A bipolar EEG montage showing neighboring potential differences enables the identification of locales where groups of neurons are firing inversely from another group of neurons at juxtaposed locales. This is a phase reversal, which concerns identifying the epileptogenic foci of spikes.

In recent years, several studies have been devised to detect seizures from EEG data by single channel EEG signals [3]–[10]. On the other hand, spikes usually occur more frequently before seizures, there are also studies in the literature focusing on the investigation of spike detection [7]–[14]. However, the seizure detection algorithm and the spike detection algorithm are usually implemented on different systems. Therefore, it is necessary to combine seizure detection and spike detection algorithms in one system for better efficiency. Furthermore, the brain is a complex and nonlinear dynamic system; it is not enough to detect seizures by a single channel EEG, which gives up the dynamic spatial information [15]–[16]. Thus, the processing of multichannel EEGs became an important issue in various areas of spatial-temporal analysis [15]. However, only a few studies have focused on multichannel EEG signals because of the challenge of effectively extracting useful information from them [16]. The analysis process of multichannel EEG is time consuming, especially for long-term EEG monitoring that typically lasts for 24 hours to several days. To process the abundant information contained in multichannel EEG data, it is necessary to have an efficient system. In addition, neurophysiologic knowledge, such as the concepts of “phase reversal” and “potential fields,” in a bipolar montage has not been applied in most previous studies, particularly in methods that determine the morphology of slow waves or spike repetition [17], [18].

To the best of our knowledge, there are no reliable computationally viable algorithms for detecting large varieties of both seizures and spikes from multichannel EEG data that operate well in clinical environments. Thus, we aim to develop a physiology-based algorithm for detecting epileptic seizures and interictal spikes from multichannel EEG signals (unipolar EEG and bipolar EEG).

**Figure 1:**
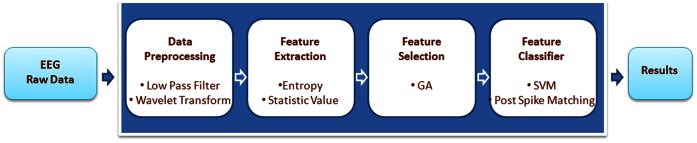
Flowchart illustrating the system architecture.

A flowchart of the system architecture is shown in Figure 1. Our EEG epilepsy classification system consists of four major components: data preprocessing, feature extraction, feature selection, and classification.

## Materials and Methods

### 2.1. Data Acquisition and Preprocessing

Clinical Data were collected from subjects receiving routine EEG examination or bedside EEG monitoring. The routine EEG of the 13 outpatients (5 women and 8 men, ages ranged from 23 to 89 years) were obtained in a clinical EEG laboratory each for around 15 minutes and were designated as Data Set I. Five subjects were in the patient group (2 women and 3 men; mean age 57.8 years old, SD = 24.4 years), they were patients with the diagnosis of temporal lobe epilepsy and they all had abnormal EEG signals. Eight were in the control group (3 women and 5 men; mean age 51.4 years old, SD = 21.27), they were adults referred from the outpatient clinic with normal EEG, they usually complained of headache or dizziness but did not have the diagnosis of epilepsy or seizure disorders. We further included five in-patients (3 women and 2 men, age ranged from 22–48 years old), which included three patients with active spikes or frequent seizures and two with non-epileptic seizure. They received bedside long-duration EEG monitoring while they stayed in the hospital for either diagnosis or treatment purpose; these were around 360 hours in total and were designated as Data Set II.

**Figure 2:**
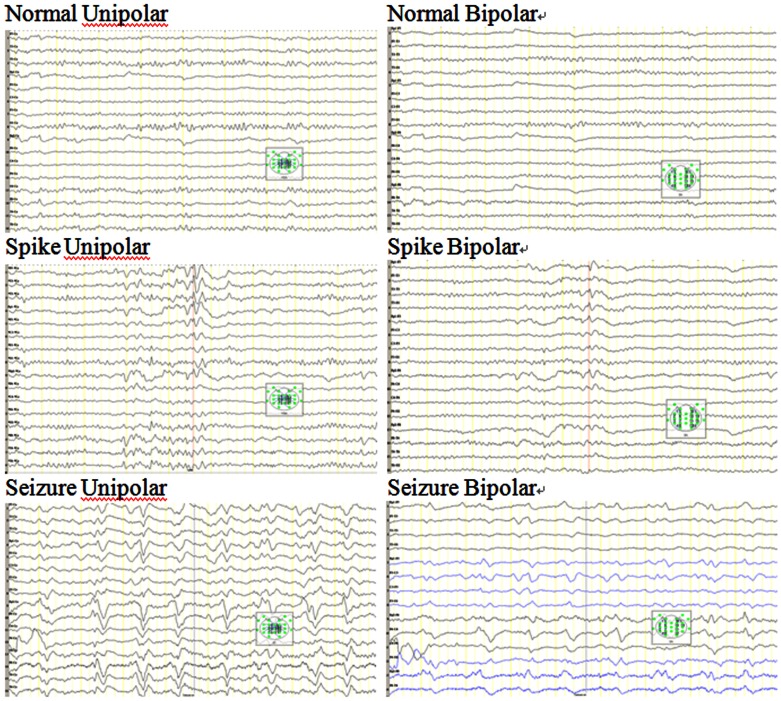
Exemplars of EEG from the three conditions, normal, spike and seizure displayed in both unipolar and bipolar montages. The right middle figure shows phase reversal of spikes in a bipolar montage.

EEGs were performed using 21 Ag-AgCl electrodes arranged on the scalp according to the 10–20 International System and were digitized at a sampling rate of 200 Hz over a dynamic range of 12 bits. Both unipolar (Figure 2, left) and bipolar (Figure 2, right) recording data were analyzed in this study. This study was approved by the ethics committee of the university hospital. All identities or personal information of the participants were delinked and were inaccessible to all researchers.

The recorded EEGs were segmented into 2-s epochs. The following criteria were used to identify inter-ictal epileptic form discharges (all designated as spike thereafter in this report for simplicity) [19]: (1) they must be paroxysmal; (2) they must include an abrupt change in polarity occurring over several seconds; (3) the duration of each transient should be less than 200 ms (i.e., spikes less than 70 ms and sharp waves between 70 and 200 ms); and (4) the discharge must have a physiology field. EEG abnormalities in patients with seizure disorders can be categorized as either specific or nonspecific patterns.

The electrographic onset of a seizure is characterized by a sudden change in frequency and the appearance of a new rhythm. Focal onset of the electrographic seizure may evolve through several phases: (1) focal desynchronization or attenuation of EEG activity (less than 10 µV); (2) focal, rhythmic, low-voltage, or fast-activity (greater than 13 Hz) discharges; and (3) a progressive increase in amplitude with slowing that spreads to a regional anatomic distribution. Epileptic seizures can be recorded as paroxysmal repetitive spikes, spike-and-wave (three or more discharges in sequence), or rhythmic fast or theta activity (all designated as seizure thereafter in this report for simplicity). Because EEG experts may have differing opinions on EEG classification, two EEG experts (Drs. Pan and Chiu) evaluated the spikes and seizures independently, and this study only used EEG segments that had a consensus for further analysis. Therefore, each 2-s epoch among the EEG data was classified into normal EEG, spikes, and seizures. All EEG data received artifact rejection prior to processing. This study removed segments with an amplitude of an EEG signal greater than 100 µV in any channel in the preprocessing stage. The EEG signals then underwent a 0.1–70 Hz band pass, a 60 Hz notch, and Daubechies 4 wavelet filters (implemented in MATLAB).

### 2.2. Feature Extraction

#### 2.2.1. Sample entropy

Sample entropy (*SampEn*) is widely used in estimating the regularity of time series data and has been applied in the processing of various biomedical signals such as heart rate variability and pulsatility of endocrine hormone release. *SampEn* quantifies the regularity of time-series data, and is called regularity statistics. It is represented by a simple index for the overall complexity and predictability of each time series. Therefore, more regular data indicate a lower value of *SampEn*. It has been used in processing EEG signals to evaluate quantitative parameters for studying the complexity of EEG signals. Experimental results have shown that *SampEn* is a useful technique for processing non-stationary signals. Among the regularity statistics, *SampEn* is robust to noisy physiological time series and is especially useful for detecting seizures or brief spikes in EEG data [20].

The calculation of *SampEn* in (1) to (3) with a signal ***S*** (finite length *N*) was performed by following step 1 through step 6. The parameter *m* was the length of the sampling window, which was the dimension of the vector to be shifted, and *r* was the value of the threshold representing the noise filter level chosen in the range of 0.1 to 0.9.

1

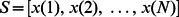
 is the vector of data sequence.
2 x*(*i*) is a subsequence of ***S*** such that 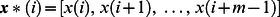 for 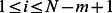
_._
3 Let *r* = *k* × SD for *k* = 0.1∼0.9 where SD is the standard deviation of ***S***.
4 For each 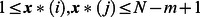, 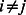, *d* [ ] is the operator of Euclidean distance.

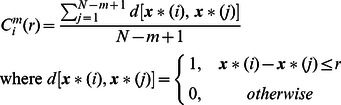

5 The quantity *A^m^*(*r*) is calculated as

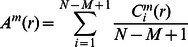

6 Finally, the *SampEn* is defined as follows:

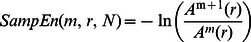



#### 2.2.2. Statistical features of unipolar EEG and bipolar EEG

Each 2-s epoch consisted of signals from all 16 channels. The four-stage wavelet transformation decomposed the filtered signal into eight frequency ranges, which included the five primary EEG frequency bands (i.e., delta, theta, alpha, beta, and gamma) used for further feature extraction [22]. Both unipolar and bipolar 16-channel EEGs were used to calculate the values of maximum, minimum, sum, average, and standard deviation (five sub-features). Then we computed five major features namely, entropy, total variation, standard deviation, sample entropy, skewness, and energy, utilizing the aforementioned 10 sub-features (five frequency bands plus five values).

**Figure 3:**
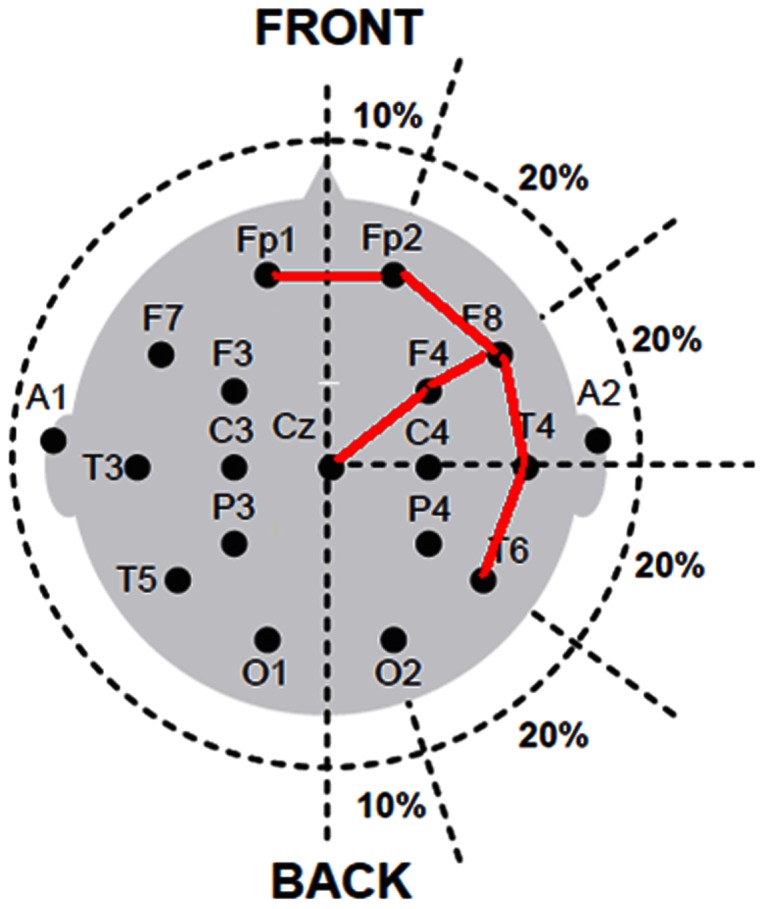
The feature extraction of the 6 neighboring channels.

Bipolar EEG has an additional process of feature extraction from phase reversal. Bipolar EEG signals were derived from the 16 unipolar channels. Each bipolar EEG channel was used to select six neighboring channels; subsequently differences of these channels were summed (as for Channel F8 in Figure 3). Numerically, phase reversal indicates the turn of the phase in juxtaposition channels, therefore the sum of the differences with the 6 neighboring channels should be minimal at a channel position of phase reversal which implies an epileptogenic focus (cortical neuron origin of spikes). Thus each major feature contains 20 sub-features from phase-reversal sum of the 16 channels plus their values of minimum, sum, average, and standard deviation. The minimum value was used to evaluate the phase reversal and local field potential change in time domain. These parameters were further used to extract the same five major features namely total variation, standard deviation, sample entropy, skewness, and energy as in unipolar and bipolar EEGs [21].

In brief, there are five major features (total variation, standard deviation, sample entropy, skewness, and energy) in our feature extraction. Both unipolar and bipolar montages have 16 EEG channels. Each major feature has 10 sub-features including five frequency bands (gamma, beta, alpha, theta, and delta) [36] and the values of maximum, minimum, sum, average, and standard deviation from all 16 channel. In total these make up 800 features (16×5×10) for both unipolar and bipolar EEG. There are additional 100 features from the computation of phase reversal in bipolar EEG. In total 1700 features were obtained from the process of feature extraction.

Total variation and standard deviation can be good discriminators of non-specific seizure activity. In addition, high energy content is an observable feature during seizures, and skewness measures the asymmetry of a distribution, which is influenced by the shape of spikes and waves. The statistical characteristics of various feature types can help distinguish different epileptic states. If there is any abnormal activity across all EEG sub-bands, then the statistical features may magnify the anomaly.

On the other hand, a bipolar EEG signal is derived from potential difference between neighboring electrodes. Neurologists and epileptologists can define spikes and other epileptiform discharges much easier by using displays from various bipolar montages. Therefore, theoretically it is possible to use a bipolar EEG independently without using wavelet transform for detecting spikes. We tested this hypothesis by extracting features from a bipolar EEG.

### 2.3. Feature Selection

A genetic algorithm (GA) is a search heuristic that generates optimizations and solutions to search problems [23]. The concept behind the GA is to mimic the natural selection process, which involves mutations, inheritance, crossover, and selection [24]. In nature, the selection process yields the fittest subjects–survivors. For search problems, such as the problem of finding optimal parameters for an operation, the selection process tests the fitness of parameters and determines optimal parameters. The GA simulates cells in nature, and its main component elements comprise genes, chromosomes, groups, and a fitness function. For each generation, the fittest cells have the best current chromosomes and are survivors. The surviving cells evolve generation to generation, attempting to become better adapted to their environment. The GA begins by creating a population of randomly generated individuals represented in binary strings consisting of 0s and 1s. The fitness of each individual is evaluated for each generation, and the fittest one is selected. Surviving individuals can mutate, recombine, and mate to generate the next genetic generation. Next, the algorithm checks whether the termination condition has been achieved. If the termination condition has not been met, the selection process continues until the termination condition is achieved.

Feature selection prior to training a support vector machine (SVM) classifier is crucial. In this study, a chromosome encoded a selected subset of features for use in SVM classification. Each generation contains 100 chromosomes, each representing a different subset of features. For each chromosome *g*, a standard binary SVM classification SVM (*p*, *q*, *g*) is invoked. To encode the subset of selected features in a chromosome, a binary code represents an individual feature. For a particular chromosome *g*, if *g*[*i*] is set to 1, the *i*th feature is included, whereas 0 indicates the exclusion of this feature [25]. The results of SVM training of individual chromosome from each GA generation were compared for their performance and 1% mutation was given to maintain genetic diversity. When the GA terminates, either reaching plateau of performance or 200th iterations, the most accurate SVM classification is chosen, as shown in Eq. (1).
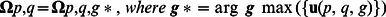
(1)


If the GA evolves *R* generations, the number of standard SVM invocations is |*M_1_*|*|*M_2_*|*…*|*M_R_*|, where |*M_k_*| is the number of chromosomes in *M_k_*.

### 2.4. Feature Classification

The two-step classification is the SVM prediction and spike matching. The components are designed separately and modularized. Within each component, further modularization occurs, meaning that each component can be used in other studies. This allows for easy upgrades and changes in the future. Another advantage of the modularized approach is that modules can be easily replaced, which could enable experimentation on the influence of a different classification technique.

#### 2.4.1. Support vector machines

The SVM maps input the featured vectors into a high-dimensional space to realize a linear classification system [26]. By feeding the algorithm a set of training data, an SVM can determine an optimal hyper-plane that minimizes risks. This study first focused on the training problems of a class pair. A training set of instance-label pairs (***x***, ***y***) and weighting vector ***w*** can be written as Eq. (2), in which ***x*** and ***y*** denote the input and output domains, respectively [27].
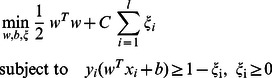
(2)


Constraint (2) has penalty term C chosen by the user to assign a penalty to errors, and *ξ* is a slack variable. It may not be useful in achieving high training accuracy (i.e., classifiers accurately predict training data with class labels that are known). Therefore, a typical method is separating training data by mapping instances into high-dimensional domains to construct models. After data are mapped into higher-dimensional spaces, the number of variables becomes large, or even infinite.

#### 2.4.2. Post-Classification spike matching

The proposed SVM classifier performed the best at distinguishing between normal and seizure activity, although further improvement regarding the spike detection rate by examining the normal outputs from the classifier was desired. Therefore, this study proposed the spike-matching method. To the best of our knowledge, no other study has used a post-classifier filter to attempt to capture epileptic spikes.

The spike phase of the spike-and-wave complex corresponds to the depolarization of the membrane potential and the repolarization and hyperpolarization constitute the wave phase. Epileptiform discharges include sharp waves, spikes, spike-and-waves, and multiple spikes and wave complexes. Sharp waves are transient and are clearly distinguishable from background activities, and have pointed peaks and durations of 70–200 ms. Spikes have a similar definition, except that the duration is 20–70 ms.

**Figure 4:**
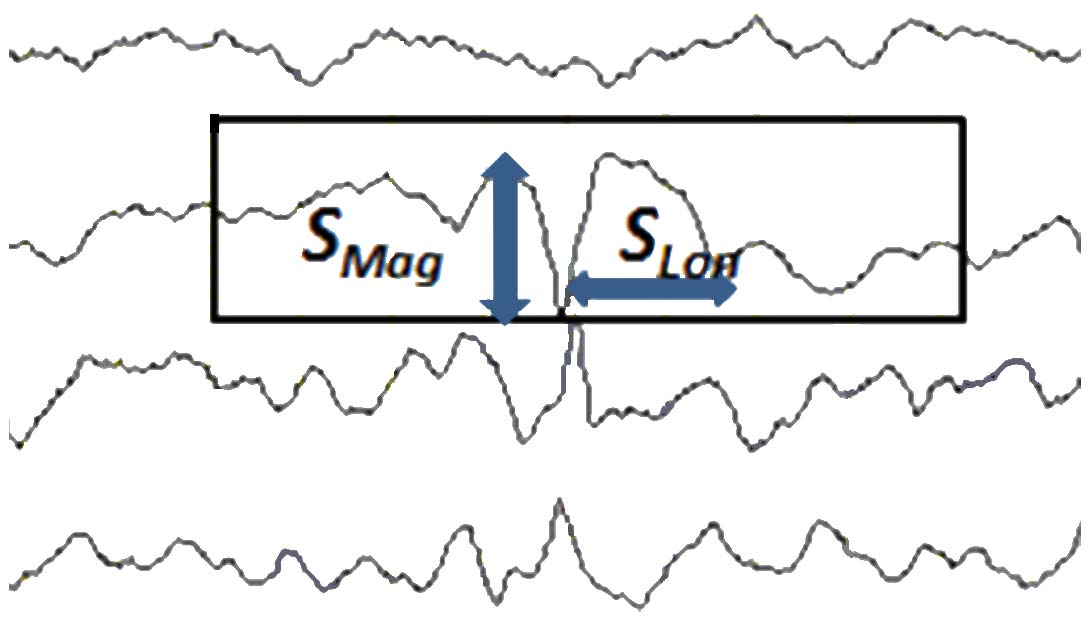
The magnitude and longevity of a burst.

The digital profile of an epileptiform discharge, such as a spike or sharp, includes the definition of the magnitude and the duration of the spike or sharp wave (Figure 4). The summed score of the magnitude and the duration of a spike or sharp wave is defined as *S_pm_* (Eq. 4), and can be tested by a trial-and-error process using a threshold value to perform post-classification spike matching.
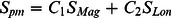
(4)


Typically, when a spike occurs, the opposite neighboring EEG readings show opposite signs in a bipolar montage. This is phase reversal. A short pulse discharge alone does not imply a spike, and neither does a phase reversal. Therefore, this study designed a spike-matching program to detect spikes in two stages. The first stage detects the short pulses of discharges. The second stage checks for phase reversals. If an EEG segment passes these two criteria, it is considered a spike. The spike-matching method alone is a poor tool for finding spike segments because spikes must be distinguished from background activities and must be matched for morphological and durational definitions. Additional uncertainties frequently arise, such as noises or pure coincidences of pointed peaks in phase reversal, which can cause a condition in which a normal EEG segment is classified as a spike.

Epileptiform discharges occur consecutively or periodically and typically have poor prognosis [28]. This type of regular and periodic discharges is called periodic lateralized epileptiform discharges (PLEDs). Therefore, consecutive or periodic epileptiform discharges pose higher risks for seizure onset. Standalone spikes pose a lower threat than do spike clusters. A 10-s EEG following a classifier-labeled spike was screened by the spike-matching program and the classifier. If either the classifier or the spike-matching program label any of the following five EEG epochs (10 s) as a spike, the corresponding epoch(s) was considered a segment containing spikes.

Therefore, when the SVM classifier recognizes an EEG epoch as a segment containing spikes, the following 10-s EEG is not only screened by the SVM classifier, but it is checked by the spike-matching block. Spike matching is more lenient than the SVM classifier. Thus, it was only used immediately following a spike being detected by the SVM. Because the classifier classifies any 10-s segment following a spike as normal background, the SVM-classified normal segment is re-examined by the post-classification spike-matching block to ensure that no spike is undetected, thereby enhancing the spike detection rate. Although this is achieved at the cost of increased false alarms, it is feasible to exchange an acceptable rate of false-positive predictions for epileptic epileptiform discharges or seizure activities for higher sensitivity in clinical practice for patients with epilepsy.

### 2.5 Statistical Evaluation

The discriminating power of the investigated technique was evaluated by constructing a classification (confusion or contingency) table for the 2-class problems with details of true positives (TP), true negatives (TN), false positives (FP), and false negatives (FN). The most frequently used evaluation measure in the performance of classification is accuracy (Acc), which is the proportion of correctly classified instances: Acc = (TP+TN)/(TP+FP+TN+FN). Other indices included sensitivity, Sen = TP/(TP+FN) and specificity, Spe = TN/(TN+FP).

## Results

### 3.1 Experimental Results

EEG of the Data I composed of three classes namely spike, seizure, and normal EEG in both unipolar and bipolar montages (Figure 2). The expert-annotated routine EEG records (Data Set I) that were obtained included 1939 two-second epochs of normal activity, 436 two-second epochs of spike activity, and 444 two-second epochs of seizure activity. The relative proportions did not reflect real-life occurrences. Spikes and seizure do not typically occur this frequently. However, these two waveforms are of the upmost detection priority, and therefore, more samples were needed for a strong prediction model. Half of the 2-s epochs were used for training, and the other half of the 2-s epochs were used for prediction. The proposed system can currently produce an output of 1700 features for each 2-s epoch. In the 360-hour bedside EEG, there were about 6 hours classified as seizures and about one hour for spikes.

We used these 1700 features together to obtain an initial classifier, which was used as a benchmark for future configurations. The recognition rates(accuracy)of spikes (Acc 83.02%) and seizures (Acc 99.77%) obtained by GA-SVM were higher than those obtained only by the SVM (Acc of spike 65.59%, Acc of seizure 95.05%). This suggests that the GA played a crucial role in the classification.

The recognition rates for seizure activities are always higher than those of spikes. Seizure activities typically last from a few seconds to a few minutes, and they display EEG patterns that are easily distinguished from background activities. The problem of classifying between normal and seizure waveforms has been partially resolved by previous studies and remains an area of ongoing research [29]. Conversely, it is more difficult to distinguish spikes from the background EEG activities using algorithms. This is not unexpected because a typical spike lasts approximately 70 ms without preceding warning signs. Our feature extraction used 2-s epochs. Therefore, statistical features can be easily masked because of the intrinsic characteristics of spikes that contribute to lower spike recognition rates.

Using the GA for feature selection, we obtained a list of the effects of various features. The list revealed the effectiveness of the features, and indicated items that were focused on for feature extraction. Table 1 shows the top five feature types, and the type of the feature and the sub-band for the feature type. A feature type represents a range of features that use the same feature extraction function. The top five feature types represented nearly 80% of the top 100 features of the 1700 features.

**Table 1:** The top-ranked feature types and associated frequency bands.

Rank	Frequency (Hz)	Sub band	Feature Type
1	0∼4	Delta wave	*SampEn*
2	4∼8	Theta wave	Total Variation
3	8∼15	Alpha wave	*SampEn*
4	8∼15	Alpha wave	Total Variation
5	4∼8	Theta wave	*SampEn*

**Figure 5:**
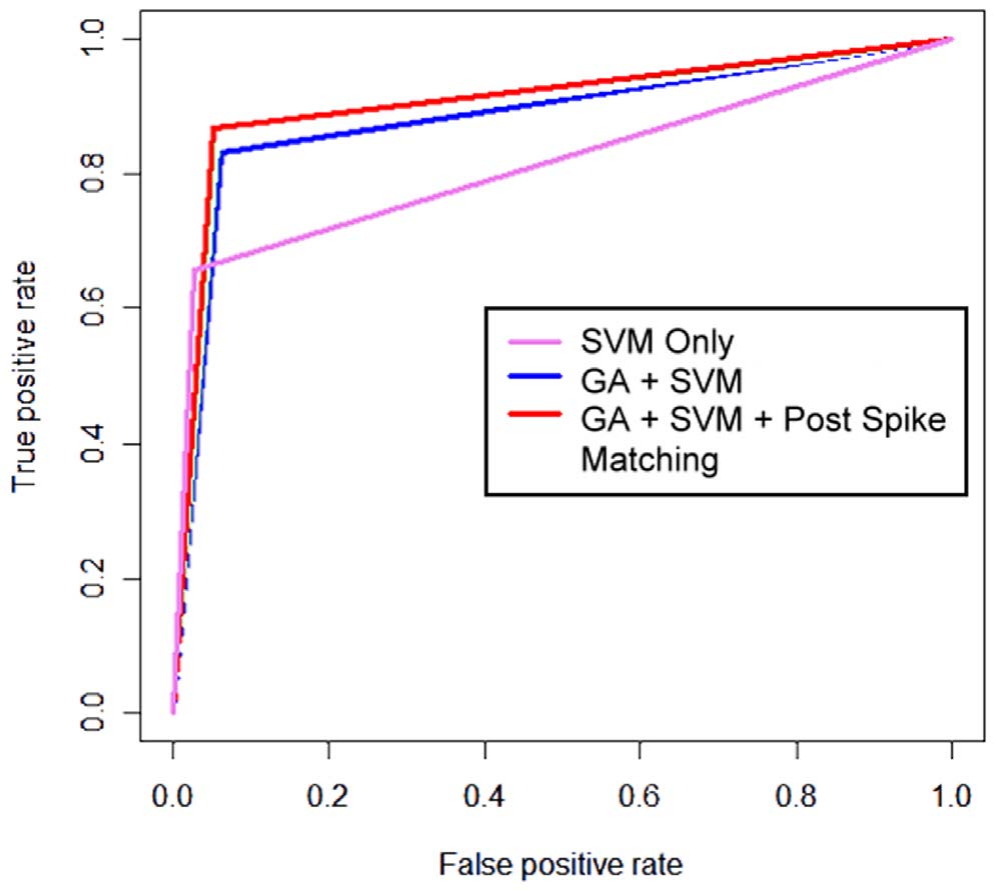
ROC Curve of different classification methods (SVM only, GA+SVM, GA+SVM+Post Spike Matching).

This study used the post-classification spike-matching program in conjunction with the best performing classifier, which used only *SampEn* and total variation-type features. Using post-classification spike-matching program further increases the recognition rate for spikes (Acc from 83.02% to 86.69%). The spike recognition rate is increased. However, it is only increased by 3.67% at the cost of a 0.51% decrease for the recognition rate of normal EEG. Receiver operating characteristic (ROC) analysis showed that the area under an ROC curve of the classifier, including the spike matching, was the largest among all of the classification methods tested in this study (Figure 5). In addition, classification only by the SVM was the worst among the four types of classifiers, indicating that the GA plays a crucial role in spike detection.

This study designed a spike-matching program to analyze bipolar montage EEG values. The spike-matching program used derivatives to measure the speed of ascent of a spike. This included the concept of phase reversal when screening for spikes [1]. When a spike occurs, the discharge spreads to its neighbors, similar to water ripples caused by a droplet. Therefore, this study used derivatives to detect phase reversals. It localizes a dipole source between two electrodes [16]. Opposite, but similar, magnitude derivatives between two electrodes implies that a spike occurred exactly equidistant from the two electrodes. An opposite but different magnitude for the derivatives between two electrodes indicates that a spike occurred between the two electrodes, but not at the midpoint of the two locations. Therefore, using a post-classification spike-matching program is a solution to improve spike localization.

To validate that the proposed EEG classification framework can be generalized to data from various acquisition machines, the classifiers were trained using all of Data Set I, and the classifiers were tested on Data Set II as a holdout data set. The classification of EEG in the Data Set II was done on continuous data. The classifier analyzed Data Set I with accuracy rates of 86.69% for spike detection and 99.77% for seizure detection. When we tested on the Data Set II, results of the spike detection rate (Acc of spike 91.18%) and seizure detection rate (Acc of 99.22%) were close to those of the performance on Data Set I. This indicates that the features analyzed in this study are stable and useful for classifying epileptiform and non-epileptiform EEG signals from patients.

## Discussion

Most seizure detection techniques currently select a few time-frequency EEG features and evaluate these features using binary thresholds [29]. To date, no single EEG feature has been identified to represent background or spikes because of numerous false positives on long-term EEG recordings [17]. The novel approach in this study is based on neurophysiologic knowledge applied by neurologists or epileptologists during their evaluation of clinical EEG in daily practice [30], [31]. The primary concept is to use the principles of field potential and phase reversal. Many neuronal elements contribute to the extracellular currents that generate field potentials recorded on the surface of the brain [30], [31], [32]. Furthermore, field potentials recorded during epileptic activity are based on changes in neuronal membrane potential. The amplitudes of epileptic field potentials exceed those of non-epileptic potentials because the underlying neuronal activity is highly synchronized [32], [33]. In the scalp EEG, each recording site (considered a node) stretches neighboring electrodes (e.g., the six nodes shown in Figure 3). According to a summation of the differences among these nodes, it is easy to display the phenomena of phase reversal. Furthermore, the proposed system added post-classification spike matching to detect spikes. Because this study attempted to find clusters of spikes that occur within short timeframes, this study was particularly meticulous in the short timeframe following a spike confirmed by the SVM classifier. When the SVM classifier recognizes an EEG epoch as a segment containing spikes, the following 10-s EEG segment is screened by the classifier and by the spike-matching block. Therefore, this method can easily integrate the time-frequency domain (wavelet coefficients) and neurophysiologic knowledge to locate spikes and seizures.

**Figure 6:**
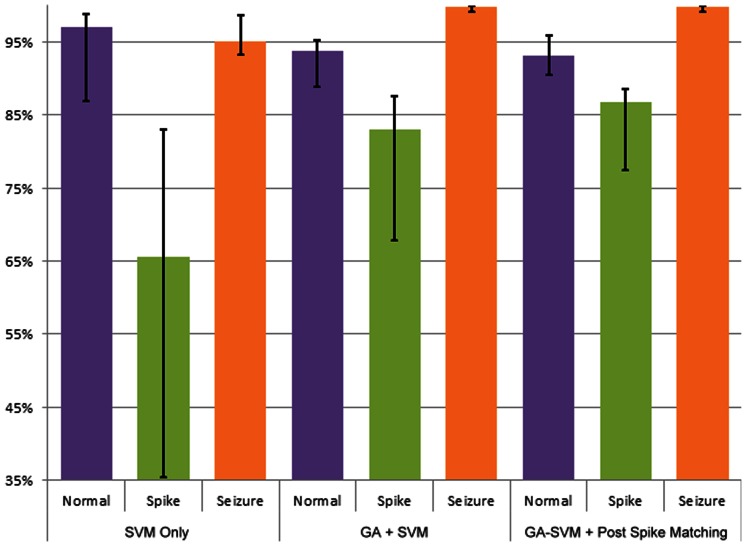
The accuracy histogram (normal, spike, and seizure) of cross validation with different methods (SVM only, GA+SVM, GA+SVM+Post Spike Matching). The trend of accuracy in normal EEG decreases slightly, but in spike EEG raises noticeably. In addition, the accuracy of seizure is stable for different feature selection and classifier.

The system was tested using EEG data from a real environment (i.e., long-term bedside EEG recordings) and yielded good preliminary results. We evaluated the proposed algorithm on the unselected, continuous, and long-term clinical monitoring of 18 patients (totaling 10,473 seizure segments from EEG recording from 363 h). The system yielded a high spike detection rate (86.69%) and seizure detection rate (99.77%) for Data Set I, which was used for model training of the system. The performance of the system in Data Set II was validated with an even higher detection rate for spikes (91.18%) and a nearly equivalent seizure detection rate (99.22%). We verified performance stability by testing Data Sets I by using the cross-validation with 10 folders, which is a technique frequently applied in Data Mining for estimating the performance of a predictive model. Data Sets I were grouped into 10 folders; and the 90% data were used as training data and the 10% holdout data were used as testing data. The train-test procedures were repeated for 10 times (10 folders). The accuracy did not vary acutely within Data Set I (Figure 6), which implies that the training models did not represent over fitting situations. Using all of the features in addition to post-classification spike matching resulted in the best outcome for detecting spikes and seizures. Therefore, from a clinical viewpoint, the automatic detection system is reliable and helpful for prescreening long-term bedside EEG data for identifying seizures and spikes.

Although the proposed method performed well for spike and seizure detection, it did so at the cost of high hardware loading and a long computation time. Typically, a 2-s segment of clinical EEG requires approximately 6 s to complete data analysis, including preprocessing, feature extraction, and data prediction. To achieve the goal of real-time processing, a parallel design for feature extraction was employed in the system that used 15 servers to collaborate in a Hadoop architecture. The consumed time was reduced to 0.5 s after applying parallel computing. The proposed physiology-based algorithm entails a quantitative method for identifying spikes and seizures that can enhance the performance of existing seizure prediction methods.

When the performance of the proposed system was compared with that of previous studies, it is among the highest rates for sensitivity of seizure detection (95.4%), spike detection (91.3%), specificity of seizure detection (95.8%), and spike detection (80.4%) (Table 2). Although all of the investigators would try to optimize the performance of their methods in their own studies, care must be taken when comparing the performance between various methods in different studies because they used different clinical data. The performance of spike detection was less than that of seizure detection, especially when the clinical EEG data obtained from the scalp surface recordings inherited all possible extracranial artifacts. Previous studies that used methods such as time-frequency analysis and template mapping were unable to simultaneously detect seizures and spikes. They were typically implemented in separate platforms. However, the proposed method provides simultaneous detection of seizure and spikes stably in a single platform. This is an additional benefit.

**Table 2:** Comparison studies in literature with our approach.

Author	Method	Prediction	Sensitivity		Specificity
Ji et al. [16]	Template method	Spike	69.3%	99.92%	
Logesparan [17]	Phase Congruency Algorithm	Spike	80%	N/A	
Lucia [18]	ICA	Spike	76%	74%	
Our study	Physiology-based Detection	Spike	91.26	80.04%	
Valder. et al. [29]	Patient Specific Algorithm	Seizure	33.38%	67.04%	
Chao. Et al. [34]	NSVM	Seizure	92%	88%	
Yadav et al. [35]	Model-Based Detection	Seizure	92.2%	100%	
Sac et al. [37]	Signal Amplitude Variation	Seizure	90.4%	N/A	
Our study	Physiology-based Detection	Seizure	95.44%	95.8%	

It is difficult to locate the occurrences of spikes and seizures by using traditional digital algorithms to fulfill a clinical diagnosis of epilepsy because EEG data contain too many complex data, especially from bedside environments. Spikes and seizures emerge infrequently and unpredictably, making it difficult for neurologists and epileptologists to identify them by using long recordings. Physicians typically cannot complete this task without using event codes recorded by patients or by their family members or caretakers. The automatic differentiation between epileptiform discharges and normal brain activity is challenging regarding the morphological patterns of EEG signals. Therefore, an automatic and accurate system of epilepsy screening can significantly reduce the cost of health care and enhance the efficiency of medical diagnoses and the treatment of patients with epilepsy.

In summary, this study overcame the diagnostic challenge of epilepsy using a cascaded system combining sample entropy for feature extraction, the GA for feature selection, the SVM for classification, and most crucial, a de novo solution based on neurophysiologic knowledge of EEG interpretation. To elaborate on the contribution of this report, there are four major points of novelty. First, we applied neurophysiologic knowledge applied by neurologists during their evaluation of clinical EEG in daily practice [30], [31]. The primary concept is to use the principle of field potential and phase reversal by evaluating potential differences of the neighboring electrodes. Second, we cascade different algorithms of feature extraction, selection, and classification [36]. Although separately these algorithms are not new ideas, the novelty stems from improved efficiency of spike and seizure recognition with the combination use of these methods. The third is the post spike matching. Spikes with great clinical significance frequently occur in tandem and tend to seizures. Thus, we use post spike matching algorithm to detect spikes after SVM classification, and accuracy of the spike detection was increased from 86.69% to 91.18%. Though the increment is not much numerically, it has a critical clinical value that we are capable of identifying those spikes with higher risk to seizures. The final novelty is the model trained from Data Set I can be generalized to work effectively on the Data Set II which were collected from an independent group of patients.
